# Acoustic Radiation Force Impulse Elastography Assessment of Lymphoedema Tissue: An Insight into Tissue Stiffness

**DOI:** 10.3390/cancers14215281

**Published:** 2022-10-27

**Authors:** Jennifer Sanderson, Neil Tuttle, Liisa Laakso

**Affiliations:** 1 School of Allied Health and Social Work, Griffith University, Gold Coast, QLD 4215, Australia; 2 School of Health Sciences, University of Tasmania, Newham, TAS 7005, Australia; 3 Mater Research Institute, The University of Queensland, South Brisbane, QLD 4101, Australia

**Keywords:** lymphoedema, ultrasound, ARFI, elastography, tissue stiffness, pitting test, pitting

## Abstract

**Simple Summary:**

Palpation is essential for clinical detection and evaluation of lymphoedema progression. As palpation sense is not quantifiable, this study investigates the use of ultrasound elastography to quantify stiffness of lymphoedema tissue and explore the influence of the pitting test on tissue stiffness. Fifteen women with unilateral arm lymphoedema were scanned using 18 MHz and 9 MHz linear transducers with a Siemens S3000 Acuson ultrasound (Siemens, Munich, Germany). Tissue stiffness comparisons were made between lymphoedema and contralateral unaffected sites, and pre-test and post-pitting test images. The novel pre-test and post-pitting test elastography method provided information of greater clinical meaning than pre-test imaging alone.

**Abstract:**

Palpation remains essential for evaluating lymphoedema to detect subtle changes that may indicate progression. As palpation sense is not quantifiable, this study investigates the utility of ultrasound elastography to quantify stiffness of lymphoedema tissue and explore the influence of the pitting test on tissue stiffness. Fifteen women with unilateral arm lymphoedema were scanned using a Siemens S3000 Acuson ultrasound (Siemens, Germany) with 18 MHz and 9 MHz linear transducers to assess tissue structure and tissue stiffness with Acoustic Radiation Force Impulse elastography. Ninety sites were assessed, three on each of the lymphoedema-affected and contralateral unaffected arms. A subgroup of seven lymphoedema-affected sites included additional elastography imaging after a 60-s pitting test. Dermal tissue stiffness was greater than subcutaneous tissue stiffness regardless of the presence of pathology (*p* < 0.001). Lymphoedematous tissue exhibited a higher dermal to subcutaneous tissue stiffness ratio than contralateral sites (*p* = 0.005). Subgroup analysis indicated that the pitting test reduces dermal tissue stiffness (*p* = 0.018) and may alter the stiffness of the subcutaneous tissue layer. Elastography demonstrates potential as a complement to lymphoedema palpation assessment. The novel pre-test and post-pitting elastography imaging protocol yielded information representative of lymphoedema tissue characteristics that could not be ascertained from pre-test elastography images alone.

## 1. Introduction

Lymphoedema is a chronic condition that presents with abnormal swelling occurring as a result of lymphatic system dysfunction [1]. Lymphoedema presents with visible swelling in localised or broad body regions, and multiple factors contribute to its severity and progression [1]. Of the many lymphoedema assessment tools used across health services, palpation (feeling with the hands and fingers) and the pitting test remain essential to clinical evaluation of lymphoedema. Palpation and the pitting test are used to detect changes in tissue characteristics (e.g., skin tightness, tissue density or signs of swelling) and tissue responsiveness in order to locate lymphoedema-affected regions, document observations of tissue texture indicative of pathological changes, and determine International Society of Lymphology (ISL) staging [1]. As a component of lymphoedema assessment, palpation is critical to forming a clinical judgement regarding underlying pathological changes of oedema accumulation, fibrotic and fatty deposition. Often patients will experience subjective and localised symptoms prior to meeting proposed diagnostic thresholds for limb volume change (a subclinical threshold of >3% change in volume from baseline measures, using a comparison with contralateral limb volume to control for weight change). Therapists often use clinical interview and palpation to diagnose lymphoedema in uncomplicated presentations [1].

The pitting test is described as an indicator of oedema presence [2]. Briefly, the pitting test involves applying sustained pressure to skin using a thumb or finger and observing whether an indentation occurs. The appearance of an indentation signifies underlying oedema of soft tissues [2]. Assessment findings often guide treatment decisions by indicating improvement or deterioration in tissue health and as indicators of treatment efficacy [2]. However, palpation and pitting test assessment is susceptible to discrepancy between therapists, with experience, skill, knowledge, sensitivity, and technique, all affecting the clinician’s ability to detect subtle changes [3,4]. Furthermore, the range of information perceived during palpation and the pitting test is not objectively quantifiable.

Elastography offers a non-invasive method to quantify tissue stiffness (as a mechanical property different to that which may be felt during palpation) and to complement lymphoedema assessment. Elastography techniques introduce a form of mechanical stress to the tissue by way of compression or shear waves, and measure the resultant tissue strain, which is the way that the tissue deforms or adapts in response to the stress applied [5]. The mechanical property of tissue stiffness is calculated using an elasticity modulus equation specific to the elastography method utilised, which is derived from stress and strain values [5]. Acoustic Radiation Force Impulse (ARFI) elastography involves the production of ultrasound-generated stress in the form of shear waves that move transversely to the direction of ultrasound waves at multiple tissue depths [6]. Mechanical tissue stiffness is related to the speed of shear wave movement through the tissue, where the shear wave velocity (SWV) is lower in soft tissues, such as skin, and higher in harder tissues, such as tendon. An elastography colour map represents the distribution of tissue stiffness using SWV data and is expected to relate to tissue characteristics associated with palpable change in tissue texture and the way the tissue responds to the pitting test. Even though the clinical meaning of tissue stiffness in lymphoedema is unclear, measuring tissue stiffness using ARFI elastography provides an objective indicator of underlying tissue properties that can be re-assessed objectively. 

Historically, a change in tissue stiffness has been associated with deterioration in tissue health [7]. Hence, elastography is emerging as a clinically useful tool to indicate changes in tissue stiffness. Elastography has demonstrated utility in evaluating connective tissue, organs, and muscle in a range of conditions to complement diagnostic assessment and as an indicator of disease progression [8]. 

Research supports clinical utility of elastography in evaluating lymphoedema. Tissue affected by lymphoedema has been shown to have higher dermal stiffness compared to unaffected tissue [9,10,11]. As a method by which to assess outcomes, elastography has also demonstrated potential to determine lymphoedema treatment effect and guide clinical reasoning. Lymphoedema tissue stiffness may increase or decrease in response to sustained localised compression, where the application of pressure is similar to the pitting test [12]. Clinical interpretation of tissue stiffness change with localised compression has been attributed to compositional content and fluid movement [12]. 

Although preliminary work supports the use of elastography in lymphoedema assessment, research is yet to guide the technique and interpretation of elastography results in everyday clinical practice; and how it might supplement the findings of the pitting test. Therefore, this study evaluates an ARFI elastography assessment protocol to quantify and observe characteristics that differentiate lymphoedema-affected from unaffected tissue and to explore tissue response to the pitting test.

## 2. Materials and Methods

The data presented in this publication has been collated from a larger study investigating characterisation of lymphoedema tissue [13] and the pitting test [14]. The participants comprised fifteen women with unilateral breast cancer-related lymphoedema, assessed as ISL stage 1 and stage 2 [1], who had completed their first-line cancer treatment and were greater than three months after completion of chemotherapy. Exclusion criteria consisted of pregnancy, pacemaker, and risk of bilateral arm lymphoedema.

All participants provided written consent. The study protocol was reviewed and approved as part of the primary author’s doctoral candidature at Griffith University, Gold Coast, Australia. As a non-intervention study, the trial was not registered. Ethical approval was sought and received from two institutions: Griffith University (GU Ref No: 2016/353) and The Centre for Advanced Imaging, The University of Queensland (UQ Ref No: 2016000887).

All participants were evaluated using ARFI elastography and high-frequency greyscale imaging pre-pitting test, and a subgroup of seven lymphoedema sites using additional elastography imaging post-pitting test. The sites tested in the subgroup were chosen throughout the data collection process as a result of researcher curiosity, and as such, the post-pitting elastography outcomes were incidental findings.

The Siemens Acuson S3000 (Siemens, Germany) ultrasound device was utilised for elastographic and greyscale imaging. Elastographic mapping and quantification were performed using a 9 MHz linear transducer. An 18 MHz linear transducer was used for high-frequency greyscale ultrasound imaging to observe the appearance of the dermal-subcutaneous tissue layer border. 

Images were obtained for ninety assessment sites, including lymphoedema-affected and contralateral unaffected sites. Three sites on each arm were marked with eyeliner pencil, including the anterior forearm and posterior forearm 15 cm proximal to the ulna styloid (seated position) and the posteromedial upper arm 30 cm proximal to the ulna styloid (prone position). The analysis included inter-image and intra-image comparisons of all participants, and tissue stiffness change post-pitting test in the subgroup of seven.

### 2.1. Assessment Protocol

A standoff was used with ultrasound gel applied to the transducer and skin for all elastography and greyscale ultrasound imaging (2 cm × 9 cm Ultrasound Gel pad, Aquaflex, Parker Laboratories, Fairfield, NJ, USA). For each location, greyscale and elastography images were captured pre-test. The pitting test was performed with firm thumb pressure for a total of 60 s, as described in previous work [14], and a post-pitting test elastography image was captured for the subgroup sites.

Tissue stiffness measures were obtained at the time of assessment by the operator placing a cursor at each region of interest (ROI) on the ARFI elastography colour map image. Each image had six ROIs distributed across the colour map, three from each of the dermal layer and subcutaneous layers. Tissue stiffness quantification in shear wave velocity (SWV; m/s) was calculated from the average of three ROIs within each tissue layer. On the elastography colour map, a higher SWV value represents higher tissue stiffness in bright green to red, and lower SWV values indicate lower tissue stiffness in shades of dark blue.

We have previously found that the border between tissue layers changes with lymphoedema [14], hence we chose to also observe and categorise the border integrity in this study. The visual appearance of the dermal-subcutaneous border observed in greyscale ultrasound imaging was expected to influence elastography-measured tissue stiffness as it was observed to be related to pitting outcomes in our previous work [14]. The ultrasound appearance of the dermal-subcutaneous border was described for each location. Three descriptors were included: a clearly defined border, slight border clarity deterioration, and evident deterioration of border integrity [13]. 

### 2.2. Statistical Analysis

Statistical analyses were performed using IBM SPSS version 25 (IBM corp., Armonk, NY, USA). A dermal:subcutaneous stiffness ratio (Dermal SWV/Subcutaneous SWV) was used to quantify differences in stiffness distribution across the tissue layers observed in elastographic colour mapping. The Wilcoxon Signed Rank Test was used to compare lymphoedema-affected and contralateral unaffected tissue stiffness, and lymphoedema-affected pre-test and post-pitting test tissue stiffness. Observations of the dermal-subcutaneous border appearance in greyscale imaging and change in stiffness between pre-test and post-pitting test elastography images are described.

## 3. Results

The participants comprised a broad demographic and a diverse range of lymphoedema history and presentations (Table 1).

Regardless of the presence of pathology, the dermis had greater tissue stiffness than the subcutaneous layer in 88 of the 90 sites assessed (*p* < 0.001). In matched comparisons, the lymphoedema-affected dermis was significantly stiffer than the contralateral dermis (*p* = 0.004) (Table 2). Stiffness of the lymphoedema-affected subcutis was inconsistent between participants with the tissue exhibiting higher or lower values compared to contralateral-unaffected tissue.

**Figure 1:**
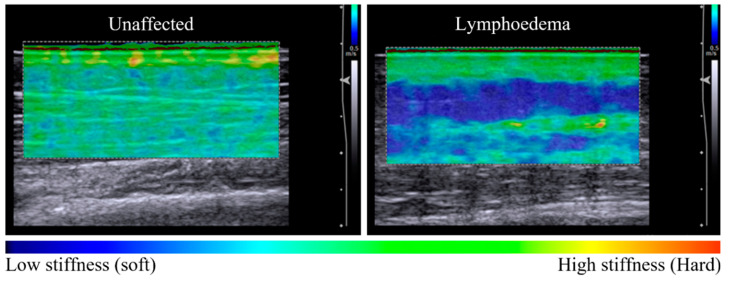
Elastography maps indicative of tissue stiffness distribution observed between unaffected and lymphoedema-affected tissue (posterior forearm, participant 11). The unaffected tissue is relatively homogenous in colour across the dermal and subcutaneous layers, with a tissue stiffness ratio of 1.38 (**left panel**). There is a difference in tissue stiffness between tissue layers of the lymphoedema-affected site with a tissue stiffness ratio of 2.69 (**right panel**). The subgroup analysis of seven sites compared elastographic imaging before and after a 60-s pitting test. In Figure 2, pre-test images for each series are similar, with a heterogenous elastographic map showing lymphoedema-affected dermal stiffness greater than subcutaneous stiffness.

Imaging indicated that lymphoedema alters the distribution of tissue stiffness across dermal and subcutaneous layers. Unaffected tissue was observed to have a relatively homogenous distribution of tissue stiffness across tissue layers in elastography maps and coincided with a low dermal:subcutaneous stiffness ratio. Lymphoedema-affected tissue exhibited higher stiffness ratio values compared to unaffected sites (*p* = 0.005), corresponding with discernibly heterogenous elastography maps (Table 2) (Figure 1).

Post-pitting test results indicate uncompressed tissue stiffness properties are altered with localised compression. Following the pitting test, the dermal tissue stiffness was lower at all sites (*p* = 0.018) while the subcutaneous stiffness either increased or decreased (not sig.) (Table 3). All tissue stiffness measures had changed from pre-test to post-pitting test, no sites or tissue layers remained the same within the test zone. 

**Figure 2:**
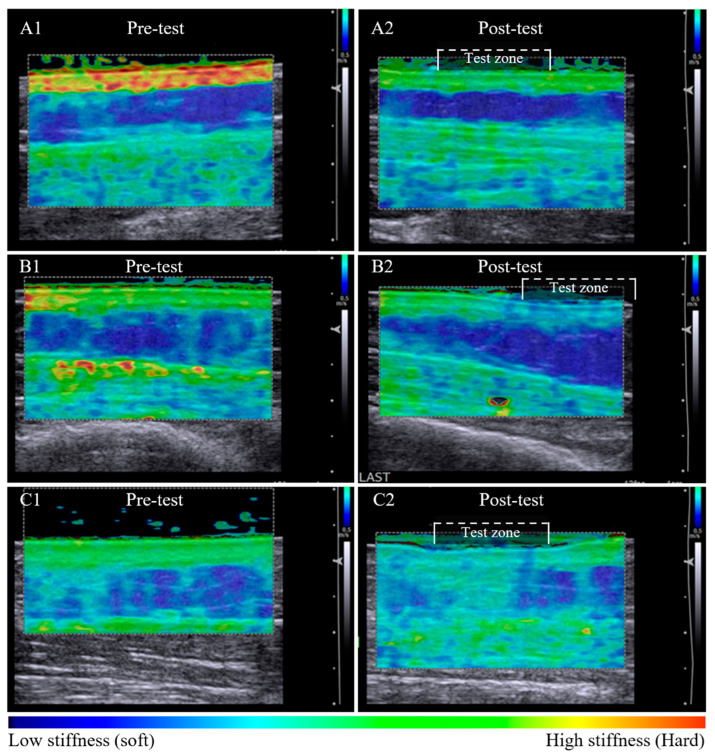
Pre-test and post-pitting test elastographic maps of lymphoedema-affected tissue. Lymphoedema-affected pre-test elastographic maps are heterogenic with high dermal stiffness (bright green) and relatively low subcutaneous stiffness (dark blue) (**A1**,**B1**,**C1**). Series A and B illustrate tissue stiffness reduction in both tissue layers post-pitting test. Stiffness is retained within the tissue layer where an intact border is apparent (**A2**), and the stiffness is altered within the test zone where there appears to be tissue border deterioration (**B2**). Tissue response was inconsistent among participants, with example (**C2**) showing a reduction in dermal stiffness and an increase in subcutaneous layer tissue stiffness post-pitting test, indicative of tissue compositional change.

Tissue stiffness changes post-pitting appeared to relate to the appearance of the dermal-subcutaneous tissue layer border. A clearly defined tissue border was associated with retention of stiffness in the dermal layer post-pitting test. The dermal:subcutaneous stiffness pattern of the test zone appears similar to adjacent tissue (Figure 2A1,A2). A less defined border was associated with a local reduction in dermal stiffness within the test zone that is visibly altered compared to the reference tissue outside the test zone (Figure 2B1,B2). Loss in border definition was also observed in tissue with subcutaneous compositional change, whereby dermal stiffness reduced and subcutaneous stiffness increased within the test zone post-pitting test (Figure 2C1,C2).

## 4. Discussion

This study evaluated an ARFI elastography assessment protocol to quantify and observe characteristics that differentiate lymphoedema-affected from unaffected tissue and to explore tissue response to the pitting test. We found that tissue stiffness across tissue layers was a differentiating feature between normal and lymphoedema-affected tissue, and the pre-test and post-pitting test imaging protocol could ascertain clinically meaningful information concerning dermal oedema, subcutaneous tissue compositional change, and structural integrity of the dermal-subcutaneous border.

Our results show dermal stiffness was consistently greater than subcutaneous stiffness regardless of whether the tissues were lymphoedematous or not. The difference in elastography stiffness values were representative of differences in tissue layer structure and density. Furthermore, healthy unaffected tissue exhibited integration of dermal and subcutaneous tissues, which manifested as a well-integrated distribution of tissue stiffness between layers. Unaffected tissue was observed to have a propensity toward homogenous elastographic maps with lower dermal:subcutaneous stiffness ratios relative to contralateral matched lymphoedema-affected tissue.

Our pre-test results agree with previous research regarding lymphoedema-affected tissue exhibiting higher dermal stiffness and variable (either higher or lower) subcutaneous tissue stiffness compared to contralateral unaffected tissue [9,10,11]. Lymphoedema alters the composition of dermal and subcutaneous tissue, which changes the distribution of tissue stiffness between and within tissue layers, as reported in the participants of this study. In pre-test imaging, we hypothesise that the increase in lymphoedema dermal stiffness is attributable to dermal free-fluid accumulation. Excess dermal free-fluid volume is thought to generate a strain on the structural fibres to accommodate the fluid. As the volume of fluid increases within the dermal tissue layer, the tissue stiffness and tension on the dermal-subcutaneous border increases, enhancing the appearance of separation between tissue layers. Correspondingly, lymphoedema-affected sites with dermal oedema also presented with evident heterogenous pre-test elastographic maps. Factors that facilitate free fluid movement might reasonably reduce dermal tissue stiffness, as shown with localised compression [15], manual lymphatic drainage [16], and the pitting test, as observed in post-pitting results.

Subcutaneous stiffness changes observed in this study corroborate earlier work by Righetti et al. (2007), who demonstrated that subcutaneous stiffness could increase or decrease with localised compression. Unlike other conditions, advancing stage and severity of lymphoedema does not result in a progressive increase in tissue stiffness [10]. Consistent with Righetti’s analysis [12], we propose the stress generated from the pitting test promotes a shift in extracellular fluid away from the test site, revealing discord in tissue stiffness within the tissue layers when compositional changes are present. We propose that any lymphoedema subcutaneous stiffness increase is due to fibrotic or fibro-fatty compositional change, and a decrease due to free-fluid or fatty composition. An increase in subcutaneous mechanical stiffness was most evident by comparing elastography maps and tissue stiffness before and after the pitting test. 

The border integrity findings have great relevance to both clinicians and researchers for understanding the effects of interventions aimed at reducing lymphoedema. The post-pitting elastography images affirm the dermal-subcutaneous border as a possible indicator of tissue structural integrity that relates to tissue deformation from the pitting test. Previous authors have described the dermal-subcutaneous tissue layer border in early lymphoedema as a clear, bright line between the tissue layers in greyscale imaging [17] and suggest the border becomes less structurally organised as lymphoedema progresses [18]. However, border deterioration is also known to occur with cellulite deposition in non-lymphoedema tissues [19]. In our previous work, we reported border deterioration to occur inconsistently across lymphoedema-affected and non-lymphoedema presentations [13]. Border deterioration is marked by a reduction in the definition of the structural line and increasing size of border undulations and serrations [19] and has been associated with greater pitting depths [14]. Although research is yet to confirm all of the factors that influence border integrity, apparent loss in border definition has been shown to be clinically meaningful with respect to pitting depth and tissue stiffness change. 

The dermal-subcutaneous border integrity was best demonstrated with pre-test and post-pitting elastography maps. An intact dermal-subcutaneous border resulted in the retention of tissue stiffness within the tissue layer boundaries in pre-test and post-pitting images. By contrast, the elastography maps of sites with border deterioration displayed reduced mechanical stiffness across tissue layers within the test zone in post-pitting images. The observation may indicate a change in structural strength of the border, however, we do not know the implications of this finding concerning tissue responsiveness to treatment, long-term outcomes, or lymphoedema progression. Further research is required to support the inference that there is clinical significance in tissue layer border integrity with respect to health outcomes.

Although the difference between lymphoedema-affected and contralateral unaffected sites suggests diagnostic capability for ARFI elastography, it would be challenging to develop diagnostic criteria for lymphoedema-affected tissue stiffness given the diversity in presentations. In addition to considerable variation in structural changes observed in lymphoedema tissue [13], the authors hypothesise that tissue stiffness is also associated with an individual’s somatotype, weight, and location of the site being assessed. Therefore, the comparative imaging method was necessary to control for multiple individual contributors to tissue stiffness, although the elastography results were more meaningful in the context of a full assessment with clinical interpretation. Future value of this assessment method may be limited to within-person assessment rather than between-person comparisons.

Our method differed from other authors with respect to the use of an ultrasound standoff to enable near-field imaging of the complete cross-section of the dermis. Preliminary testing indicated that the weight of the standoff generated a small amount of mechanical strain in the underlying tissue and slightly increased SWV. As all tissues were subjected to the same conditions, the effect of the standoff weight was initially thought to be inconsequential to tissue stiffness outcomes and consistent across participants. However, on review of pilot work without the standoff, it was noted that the stress generated from the standoff promoted mechanical strain differences between tissue layers that would otherwise have been less evident.

Although the subgroup numbers were small, the elastography method applied to these sites gleaned quantitative results representative of palpable texture and tissue responsiveness to pitting assessment. Pre-test and post-pitting elastography comparisons provided confirmation of dermal oedema movement with compression and new knowledge on tissue stiffness within and between tissue layers enabling inference of lymphoedema tissue changes and dermal-subcutaneous border function. This data would complement palpation and pitting assessment as a representation of tissue properties underlying the skin surface and as an objective determinant of treatment effect. 

ARFI elastography is an emerging technology for the objective assessment of lymphoedema. There are few validation studies of elastography, all consisting of low numbers. Nevertheless, it has been shown to have good interrater reliability (Cohen’s Kappa = 1) and test–retest agreement [20]. A more recent systematic review of elastography for lower limb lymphoedema concluded “*Our systematic review has shown that UE* [ultrasound elastography] *appears to be a great tool in the assessment of LEL* [lower extremity lymphoedema] *in moderate-to-advanced stages of disease*” [21]. One of the main limitations highlighted in our use of ARFI was in identifying a repeatable application protocol especially the amount of pressure applied by the operator and stand-off. This factor could be especially problematic in other studies over areas of pain or superficially underlying bony anatomy. The authors recognise that further research is required to refine the ARFI elastography lymphoedema assessment protocol described herein, to guide the interpretation of tissue stiffness outcomes, and determine if our suppositions regarding tissue structure and lymphoedema composition are correct. To reduce the risk of bias, future studies of ARFI should include an image assessor who is blinded to image acquisition factors. 

## 5. Conclusions

ARFI elastography demonstrates potential as a complement to lymphoedema palpation and pitting assessment to better inform clinical reasoning and as a determinant of treatment effect. Using ARFI, we showed that a pre-test and post-pitting test method could ascertain clinically meaningful information concerning dermal oedema movement, subcutaneous tissue compositional change presence, and dermal-subcutaneous border structural integrity.

## Figures and Tables

**Table 1:** Participant demographics for whole group (*n* = 15).

Demographics	Values	
	Range	Mean
Age	39–70 years	58.8 years
Lymphoedema duration	3 months–10 years	4 years
Volume		
- Affected limb	2051–3962 mL	2825 mL
- Unaffected limb	1627–3111 mL	2494 mL
Surgery		
- Lymph nodes removed at surgery	1–34	16.2
- Number of positive lymph nodes	0–16	4.0
Post-Surgical Complications	*n*	Percentage
- -Cording/Axillary web syndrome	7	46.7
- -Drainage >10 days	6	40
- -Seroma requiring aspirations	1	6.7
Cellulitis		
- Yes	1–10 episodes	26.7
- No	0 episodes	73.3

**Table 2:** Ultrasound elastography tissue stiffness of lymphoedema-affected and unaffected tissue.

Tissue Layer	Affected SWV		Unaffected SWV		Sig.
	Range	Mean (SD)	Range	Mean (SD)	*p*-value #
Dermis	1.373–5.150	3.054 (0.784)	1.757–4.540	2.851 (0.726)	0.004 *
Subcutis	0.833–3.290	1.707 (0.535)	0.847–3.550	1.895 (0.603)	0.092
Stiffness ratio	0.996–3.567	1.907 (0.642)	0.976–2.605	1.592 (0.391)	0.005 *

Note. SWV = Shear Wave Velocity m/s. SD = Standard Deviation. # Wilcoxon Signed Ranks Test. * Sig. Statistically significant comparison to the 0.05 level.

**Table 3:** Lymphoedema-affected subgroup results comparing pre-test and post-pitting test elastography tissue stiffness (*n* = 7).

Tissue Stiffness	Dermal SWV		Subcutis SWV		D:S Ratio
	Range	Mean (SD)	Range	Mean (SD)	Mean
Pre-test	2.373–5.150	3.751 (0.833)	1.040–2.331	1.677 (0.385)	2.362
Post-pitting	2.030–3.360	2.564 (0.447)	0.897–2.350	1.621 (0.620)	1.843
Difference	0.343–1.790	1.187 (0.457)	−0.897–1.284	0.056 (0.836)	
Sig. (*p*-value) #	0.018 *		0.866		0.310

Note. SWV = Shear Wave Velocity m/sec. SD = Standard Deviation. Difference = Pre-minus post-SWV. D:S ratio = Dermal/Subcutaneous SWV. # Wilcoxon Signed Ranks Test, * Statistically significant comparison to the 0.05 level.

## Data Availability

Data available on request due to restrictions associated with PhD thesis under examination and supplementary articles submitted for publication yet to be published. The data presented in this study are available on request from the corresponding author J.S.
